# Neuromuscular control strategies during the Tai Chi heel-kick movement across different levels of motor experience

**DOI:** 10.3389/fphys.2026.1873507

**Published:** 2026-09-17

**Authors:** Ze Huang, Junyu Gao, Luming Chen, Xiaoshan Lei

**Affiliations:** 1 Department of Physical Education, Wuhan Polytechnic University, Wuhan, China; 2 School of Physical Education, Chongqing University of Posts and Telecommunications, Chongqing, China; 3 Department of Social Sports, School of Sports and Leisure, Guangdong Ocean University, Zhanjiang, China; 4 College of Physical Education and Sports, Beijing Normal University, Beijing, China

**Keywords:** Co-contraction, heel-kick, Joint coordination, Muscle Synergy, spinal motor control, Tai Chi

## Abstract

**Objective:**

This study aimed to examine differences in neuromuscular control strategies during the Tai Chi heel-kick movement between practitioners with different levels of motor experience by integrating estimated spinal segmental motor output, muscle synergy analysis, antagonist muscle co-contraction, and joint coordination assessment.

**Methods:**

Sixteen male Tai Chi practitioners were divided into a Master-level group (n = 8) and a first-class group (n = 8) according to official Chinese athlete certification. Kinematic, kinetic, and surface electromyography data were synchronously collected using a Vicon motion capture system, Kistler force platform, and wireless surface electromyography system. Estimated spinal segmental motor output, muscle synergy number and structure, co-contraction index (CI), center of activity (CoA), full width at half maximum (FWHM), and joint coordination parameters based on continuous relative phase (CRP) were quantified.

**Results:**

Descriptive group-average profiles showed different temporal and rostrocaudal patterns of estimated lumbosacral motor output between groups. The participant-level mean number of muscle synergies did not differ significantly between the Master-level and first-class groups (3.73 ± 0.37 vs. 3.63 ± 0.33, p = 0.499). Three representative group-level synergy modules were identified in both groups, with module-specific differences in muscle-weighting composition. The CoA of matched M1 occurred significantly earlier in the Master-level group than in the first-class group (p = 0.004), whereas the CoA of M2 and M3 and the FWHM of all three modules did not differ significantly between groups. None of the four lower-limb co-contraction indices differed significantly between groups. Phase-level coordination analysis showed a more in-phase but more variable supporting-limb hip–knee pattern during push-off and a more in-phase and less variable kicking-limb hip–knee pattern during recovery in the Master-level group.

**Conclusion:**

Greater motor experience was associated with selective and phase-specific differences in neuromuscular organization rather than with a general reduction in synergy number or co-contraction. The findings were characterized primarily by module-specific activation timing and phase-dependent hip–knee coordination.

## Introduction

Tai Chi, a traditional Chinese mind–body exercise, integrates physical movement and mental regulation. Its slow and coordinated movement patterns have been shown to improve balance in older adults, enhance neuromuscular control, and reduce the risk of falls (Hong and Li, 2007). In recent years, advancements in biomechanics and neuroscience have encouraged researchers to examine the motor control mechanisms underlying Tai Chi through muscle activation patterns, joint coordination, and central nervous system regulation.

Among its various movements, the “heel-kick” is particularly demanding. This maneuver requires practitioners to execute a rapid and forceful lower limb extension while maintaining single-leg support, upright trunk posture, dorsiflexed foot, and precise control of body balance. These demands place high requirements on trunk stability, lower limb coordination, and the precision of spinal motor output (Huang et al., 2009). As a representative leg technique in Tai Chi, the heel-kick is critical not only for the movement’s offensive and expressive qualities but also for smooth transitions, whole-body coordination, and rhythmic control. It is therefore regarded as a key indicator of technical proficiency in Tai Chi (Ma and Jennings, 2021).

Previous studies have shown that long-term Tai Chi training can shape neuromuscular function, including enhanced functional brain connectivity (Chen et al., 2025) and spinal or supraspinal adaptations related to balance control (Taube et al., 2008). For example, Wu and Ren (2009) reported that the slower movement speed in Tai Chi promotes sustained isometric activation of knee extensors, such as the rectus femoris. This activation pattern may facilitate postural control and improve resistance to perturbation. Zhao et al. (2023) further demonstrated that high-level practitioners exhibit superior joint-pair coordination and variability regulation in typical Tai Chi movements, reflecting a more stable dynamic control strategy.

Current research on the heel-kick movement has mainly focused on biomechanical outputs, such as joint angles and plantar pressure distribution. For instance, Chen et al. (2017) highlighted the role of ankle–hip strategies in maintaining balance during the kicking phase. Huang et al. (2009) further identified the importance of feedforward muscle activation sequencing in optimizing movement efficiency. However, these findings are largely descriptive of output-level phenomena and do not reveal the underlying neuromuscular control strategies. Given that motor control is fundamentally a neural coordination process under biomechanical constraints, surface-level muscle activation changes are likely governed by deeper neural modules (Latash et al., 2002). Yet, a systematic understanding of the multilevel neuromuscular control mechanisms in complex Tai Chi movements, such as the heel-kick, remains lacking.

In addition, expertise-related neuromuscular and biomechanical adaptations have also been reported in other martial arts and kicking-based disciplines. For example, studies comparing novice and advanced taekwondo athletes have shown differences in muscle activation patterns during roundhouse kicking (Valdés-Badilla et al., 2018). These findings suggest that training experience may influence the timing and magnitude of lower-limb muscle recruitment. Research on elite karate practitioners has also demonstrated skill-dependent neuromuscular control characteristics during front-kick execution, including different activation patterns of knee flexor and extensor muscles compared with amateur practitioners (Sbriccoli et al., 2010). Furthermore, biomechanical analysis of the martial arts high front kick has shown that effective kicking performance requires coordinated lower-limb segment motion and muscle activity (Sørensen et al., 1996). These findings suggest that expertise-related differences in the Tai Chi heel-kick may not only be reflected in external kinematic performance, but also in the organization of multilevel neuromuscular control strategies.

To address this gap, the present study combines three analytical approaches—spinal segmental motor output modeling, muscle synergy analysis, and joint coordination assessment—to explore the neuromuscular control strategies of the Tai Chi heel-kick. These methods collectively examine central neural drive, peripheral muscle activation, and multi-joint coordination, offering a comprehensive perspective on motor control across hierarchical levels.

## Materials and methods

### Participants

A sensitivity power analysis was performed using G*Power 3.1.9.2. For a two-tailed independent-samples t-test with a total sample size of 16 participants, an allocation ratio of 1:1, an alpha level of 0.05, and statistical power of 0.80, the detectable effect size was Cohen’s d≈1.51. Sixteen male Tai Chi practitioners were recruited and divided according to official Chinese athlete certification into a Master-level group (n = 8) and a first-class group (n = 8). Participants in the Master-level group held Chinese national Master-level athlete certificates, whereas participants in the first-class group held Chinese national first-class athlete certificates. Athlete certification was used as the grouping criterion because it represents officially recognized competitive achievement and sport-specific technical proficiency within the Chinese athlete grading system. The master-level group had a mean age of 20.00 ± 3.83 years, height of 173.00 ± 4.55 cm, and body mass of 66.17 ± 7.24 kg. The first-class group had a mean age of 17.67 ± 1.70 years, height of 173.17 ± 6.04 cm, and body mass of 60.50 ± 3.25 kg.

Prior to testing, all participants and, where applicable, their parents or legal guardians were fully informed of the study objectives, experimental procedures, potential risks, and their right to withdraw from the study at any time without penalty. Written informed consent was obtained from all adult participants. For participants younger than 18 years at the time of data collection, written informed consent was obtained from a parent or legal guardian together with written assent from the participant. The study was approved by the Medical Ethics Committee of Wuhan Polytechnic University and was conducted in accordance with the Declaration of Helsinki.

### Data acquisition

Kinematic, kinetic, and surface electromyography (sEMG) data were synchronously collected during the execution of Tai Chi heel-kick movement (**Figure 1**). A Vicon motion capture system (T40, Vicon, UK) was used with a sampling frequency of 200 Hz. Ground reaction forces were recorded using a Kistler 3D force platform (9260AA6, Kistler, Switzerland) at 1500 Hz. Surface EMG signals were acquired using a Noraxon wireless system, fully synchronized with the motion and force data streams.

**Figure 1:**
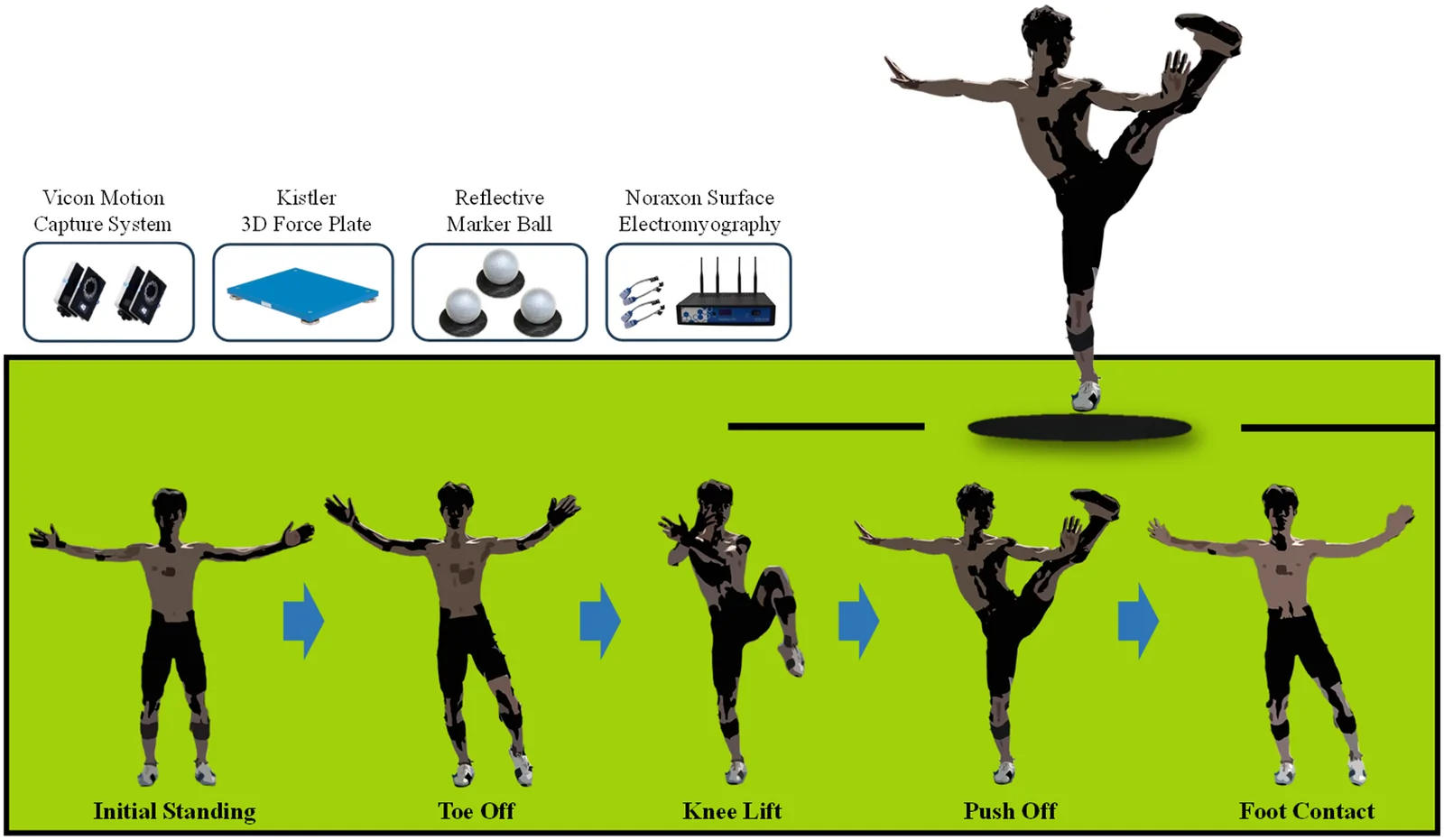
Experimental setup and schematic of the heel-kick movement.

A customized full-body model with 38 reflective markers was developed based on the default Visual 3D template. Marker placement included the forehead and occipital regions (LHAD, RHAD, LFAD, RFAD), trunk landmarks such as the clavicle (CLAV), C7, T10, xiphoid process (STRN), bilateral anterior superior iliac spines (LASIS, RASIS), iliac crests (LICST, RICST), and posterior superior iliac spines (LPSIS, RPSIS). Lower limb markers were placed on the greater trochanters (LTROC, RTROC), lateral thighs (LTH1–2, RTH1–2), femoral epicondyles (LMEP, LLEP, RMEP, RLEP), lateral shanks (LSK1–2, RSK1–2), malleoli (LMME, LLME, RMME, RLME), heels (LHEEL, RHEEL), and the first and fifth metatarsal heads (LHM1/5, RHM1/5).

Surface electromyography (sEMG) was recorded bilaterally from six lower-limb muscles, yielding 12 muscle channels in total: the rectus femoris (RF), vastus lateralis (VL), tibialis anterior (TA), biceps femoris (BF), medial gastrocnemius (MG), and gluteus maximus (GMax). These muscles were selected to represent the major superficial lower-limb muscles involved in knee-lift control, kicking-limb extension, force generation, limb recovery, single-leg support, and foot-position control. The bilateral recordings also provided the antagonist muscle pairs required for the co-contraction analysis and the muscle activity used to estimate lumbosacral segmental motor output.

These muscles were selected because they represent the major superficial lower-limb muscles involved in the main functional components of the Tai Chi heel-kick movement, including knee-lift control, kicking-limb extension, push-off force generation, limb recovery, single-leg support, and foot-position control. The rectus femoris and vastus lateralis mainly reflect knee extension and lower-limb support control; the biceps femoris and gluteus maximus reflect posterior-chain recruitment during hip extension, limb withdrawal, and post-kick stabilization; the medial gastrocnemius reflects ankle plantarflexion during push-off; and the tibialis anterior reflects ankle dorsiflexion and foot-position control. These muscles also provided the antagonist muscle pairs used for co-contraction analysis and covered the main lumbosacral segments required for estimating spinal segmental motor output. Muscles such as the iliopsoas, hip adductors, gluteus medius, and soleus were not recorded because the present protocol focused on major superficial muscles that can be reliably measured using surface electromyography.

### Movement-cycle and phase definition

The Tai Chi heel-kick movement consisted of five sequential positions: initial standing, kicking-foot toe-off, knee lift, push-off, and subsequent foot contact, as illustrated in **Figure 1**. The initial standing position represented the preparatory posture before movement initiation and was not included in the time-normalized movement cycle. The analyzed heel-kick movement cycle extended from kicking-foot toe-off to subsequent foot contact.

Four movement events were used to delimit the movement cycle and its three phases. Event 1 (E1), kicking-foot toe-off, was defined as the first frame in which the kicking foot completely lost contact with the ground, as identified from the kicking-foot marker trajectories and confirmed using the synchronized video recording. Event 2 (E2), knee lift, was defined as the frame at which the knee joint of the kicking limb reached its maximum flexion angle. Event 3 (E3), push-off completion, was defined as the frame at which the knee joint of the kicking limb reached its maximum extension during the heel-kick. Event 4 (E4), foot contact, was defined as the first frame in which the kicking foot re-established contact with the ground after limb withdrawal, as identified from the kicking-foot marker trajectories and confirmed using the synchronized video recording.

Accordingly, the knee-lift phase was defined as the interval from kicking-foot toe-off to maximum knee flexion of the kicking limb (E1–E2). The push-off phase was defined as the interval from maximum knee flexion to maximum knee extension of the kicking limb (E2–E3). The recovery phase was defined as the interval from maximum knee extension to subsequent ground contact of the kicking foot (E3–E4). The same event-detection criteria and phase boundaries were applied consistently to the estimated spinal segmental motor output, muscle synergy, and joint coordination analyses.

### Experimental procedure

Participants were first informed of the experimental procedures and precautions before providing written informed consent. Basic demographic data were recorded, and key anthropometric measurements were obtained, including trunk, upper-arm, forearm, thigh, shank, and foot segment lengths. Based on the Visual 3D full-body model, 38 reflective markers were firmly attached to the skin using double-sided tape. To minimize soft tissue artifact, all participants wore standardized compression pants provided by the laboratory, and the same technician performed all marker placements. Each sensor was checked for secure attachment, and all EMG electrodes were verified for proper channel connections. Prior to EMG data collection, the skin over each target muscle was cleaned with 75% alcohol and shaved as needed to ensure signal quality. Maximum voluntary contraction (MVC) tests were then conducted to normalize EMG signals.

Before the formal trials, a static calibration trial was recorded, followed by a 5-minute warm-up to reduce the risk of muscle strain during testing. During formal data collection, all participants were instructed to perform five trials of the Tai Chi heel-kick movement using the right limb under standardized verbal instructions. The right limb was therefore defined as the kicking limb, whereas the left limb was defined as the supporting limb. Limb use was standardized across all participants and all valid trials. The entire session was video recorded. An experienced Tai Chi coach monitored the trials on site to ensure that the movement was performed according to the required technical standard. Trials with obvious execution errors, loss of balance, use of the incorrect kicking limb, or incomplete movement sequences were excluded and repeated.

### Data processing

#### Spinal segmental motor output

For subsequent analyses, the heel-kick movement cycle was divided into three phases: the knee-lift phase, the push-off phase, and the recovery phase. The knee-lift phase represented the initial lifting and positioning of the kicking limb; the push-off phase represented the main force-generation and kicking extension period; and the recovery phase represented the return and post-kick stabilization period. These phase labels were used consistently for spinal segmental output, muscle synergy, and joint coordination analyses.

To characterize the spatiotemporal organization of total motor output, the recorded mean EMG activity profiles were mapped onto the rostrocaudal distribution of spinal motoneuron (MN) pools. This approach provides a segment-level interpretation of motor pool activation, rather than analyzing activity solely at the individual muscle level (Faul et al., 2007; Wenger et al., 2016). In this study, the segmental innervation chart proposed by Kendall et al. (2005) was used as a reference. Typically, each muscle is innervated by multiple spinal segments, and conversely, each segment contributes to the control of several muscles. To reconstruct the motor output profile of a given spinal segment Sj, all rectified EMG waveforms corresponding to muscles innervated by that segment were averaged using a weighted summation, as described by La Scaleia et al. (2014) (Equation 1):

(1) $$S_{j}=\frac{\sum_{i=1}^{m_{j}}(\frac{k_{ji}}{l_{i}}\cdot EMG_{i})}{\sum_{i=1}^{m_{j}}\frac{k_{ji}}{l_{i}}}\cdot MN_{j}$$

$EMG_{i}$represents the muscle activation envelope for a specific participant, expressed in microvolts (μV). The coefficient $k_{ji}$ is a weighting factor: it equals 1 if spinal segment j is the primary source of innervation for muscle i, and 0.5 if it is a secondary source. $m_{j}$​ denotes the number of muscles innervated by segment j, and $l_{i}$ is the total number of spinal segments (both primary and secondary) contributing to the innervation of muscle i. $MN_{j}\;$indicates the number of motoneurons within segment j. To visualize the continuous rostrocaudal spatiotemporal organization of the estimated spinal segmental output, group-average temporal profiles and filled contour plots were generated from the resulting activation matrices. The temporal profiles and contour maps were used for descriptive comparison between groups.

#### Muscle synergy extraction

All EMG data were processed using custom MATLAB scripts. The raw EMG signals were high-pass filtered at 50 Hz to attenuate low-frequency movement artifacts, full-wave rectified, and then low-pass filtered at 20 Hz to obtain the EMG envelope (Santuz et al., 2017). The processed EMG signals were then time-normalized over the heel-kick movement cycle before muscle synergy extraction.

Muscle synergies were extracted from each trial of each participant using non-negative matrix factorization (NMF) (Kargo and Nitz, 2003)^-^ (Safavynia et al., 2011). For each trial, the processed EMG matrix D(t)was decomposed into a set of time-invariant muscle weighting vectors $W_{i}$and corresponding time-varying activation coefficients $C_{i}(t)$ (Equation 2):

(2) $$\text{D}(t)\approx \sum_{i=1}^{N_{syn}}C_{i}(t){\text{W}}_{i}$$

where $N_{syn}$represents the number of synergies selected at the individual-trial level.

The number of synergies was determined separately for each trial using the variance accounted for (VAF) criterion. Candidate synergy ranks were tested sequentially, and the smallest rank satisfying both a total VAF of at least 0.90 and a minimum muscle-specific VAF of at least 0.80 was retained as N_syn_. To reduce sensitivity to the random initialization of NMF, the decomposition was repeated multiple times for each candidate rank, and the solution with the highest reconstruction quality was retained. The number of synergies selected for each individual trial is reported in **Supplementary Table 1**.

VAF was calculated as follows (Equations 3, 4):

(3) $$VAF=1-\frac{SSE}{SST}$$

(4) $$SST=\underset{i,j}{\sum }{\left(D_{ij}-mD_{i}\right)}^{2},\;SSE=\underset{i,j}{\sum }{\left(D_{ij}-{\left[WC\right]}_{ij}\right)}^{2}$$

where SSE is the sum of squared reconstruction errors and SST is the total sum of squares of the original EMG data. The minimum number of synergies satisfying a total VAF of at least 90% and a minimum muscle-specific VAF of at least 80% was selected for each trial. The NMF update process was terminated when the change in VAF between successive iterations was less than 0.001% for 20 consecutive iterations. If the maximum number of iterations was reached before this criterion was satisfied, the extraction was also terminated.

After individual-level synergy extraction, the muscle weighting vectors obtained from all trials and participants were pooled separately for the high-level and first-class groups. To identify group-level representative synergy modules, k-means clustering was applied to the pooled muscle weighting vectors within each group. Therefore, $N_{syn}$ represents the number of synergies required for EMG reconstruction at the individual-trial level, whereas the cluster number *k* represents the number of group-level representative synergy modules.

The k-means clustering was performed using the k-means function in MATLAB with squared Euclidean distance as the metric. Initial cluster centroids were randomly selected from the dataset. Each clustering process was repeated 1,000 times using different initial centroids, and the result with the lowest total within-cluster sum of squares was retained. The optimal number of representative clusters was determined using gap statistics. For the reference datasets, 500 simulated datasets were generated by uniform sampling within the bounds of the original synergy dataset. For each reference dataset, k-means clustering was repeated 100 times with candidate cluster numbers ranging from 2 to 20. The final number of group-level representative modules was defined as the smallest *k* satisfying the one-standard-error criterion (Equation 5):

(5) Gap(k)≥Gap(k+1)−sd(k+1)

where Gap(k)represents the gap statistic for *k* clusters, and sd(k+1)denotes the standard deviation of clustering compactness for k+1clusters within the reference datasets. This procedure identified three group-level representative synergy modules in both groups.

To assess intergroup differences in muscle activation duration and basic activation timing, the full width at half maximum (FWHM) was calculated. FWHM was defined as the total duration during which the activation profile, after subtracting its minimum value within the normalized movement cycle, exceeded half of its peak amplitude (Cappellini et al., 2016). To quantify the timing of muscle activation bursts and synergy activation patterns, the center of activity (CoA) was computed using circular statistics (Landler et al., 2018). CoA and FWHM were calculated from the 500-point normalized synergy activation profiles for each trial and then averaged across trials for each participant.

For CoA calculation, each time point of the normalized movement cycle was mapped onto a circular angle from 0 to 2π (Equation 6):

(6) $$\theta_{t}=2\pi (t-1)/T$$

For a given activation profile $P_{t}$, the horizontal and vertical components were calculated as (Equations 7, 8):

(7) $$A=\sum_{t=1}^{T}(\cos\theta_{t}\cdot P_{t})$$

(8) $$B=\sum_{t=1}^{T}(\sin\theta_{t}\cdot P_{t})$$

Equation 9:

(9) CoA = atan2(B, A)

where T=500, $P_{t}$represents the activation magnitude at time point t, and $\theta_{t}$represents the circular angle corresponding to that time point. The use of atan2(B,A)ensured that the resulting CoA angle was assigned to the correct quadrant.

To characterize the complementary dynamics of antagonistic muscle activation, the co-contraction index (CI) was calculated. CI quantifies the level of simultaneous activation between antagonistic muscle pairs. In this study, CI was computed for the thigh muscle pair, rectus femoris and biceps femoris, and the shank muscle pair, medial gastrocnemius and tibialis anterior. The calculation followed the approach proposed by Rudolph et al. (2000) and Martino et al. (2014). At each time point, $EMG_{H}(j)$and $EMG_{L}(j)$represent the higher and lower normalized activation levels, respectively, of the two antagonistic muscles. The CI was calculated as (Equation 10):

(10) $$\text{CI}=\frac{1}{T}\overset{T}{\underset{j=1}{\sum }}[\frac{EMG_{H}(j)+EMG_{L}(j)}{2}\cdot \frac{EMG_{L}(j)}{EMG_{H}(j)}]$$

where T=500. This method estimates the relative simultaneous activation between antagonistic muscle pairs at each sampling point and quantifies the magnitude of muscle co-contraction throughout the normalized movement cycle. A higher CI value indicates greater simultaneous activation of the antagonistic muscle pair. A lower CI value may indicate either low activation in both muscles and high activation in one muscle accompanied by low activation in the other.

#### Joint coordination analysis

Three-dimensional marker trajectories were reconstructed using the Vicon motion capture system, and joint kinematics were calculated in Visual3D using the customized full-body model. Hip, knee, and ankle joint angles and angular velocities were low-pass filtered using a fourth-order Butterworth filter with a cutoff frequency of 15 Hz. The processed joint-angle and angular-velocity data were subsequently exported to MATLAB for joint coordination analysis. For joint coordination analysis, each heel-kick movement cycle was time-normalized to 0–100% using spline interpolation. Phase angles were derived from the flexion–extension angular displacement and angular velocity of the hip, knee, and ankle joints, and continuous relative phase (CRP) was calculated from the phase-angle difference between the two joints within each joint pair (Lukšys et al., 2021).

Because the right limb was standardized as the kicking limb and the left limb as the supporting limb, CRP was calculated separately for four joint pairs: supporting-limb hip–knee, supporting-limb knee–ankle, kicking-limb hip–knee, and kicking-limb knee–ankle (Equation 11):

(11) $$CRP(i)={\text{Φ}}_{hip}(i)-{\text{Φ}}_{knee}(i)$$

Here, ${\text{Φ}}_{\text{hip}}(\text{i})$ and ${\text{Φ}}_{\text{knee}}(\text{i})$ represent the phase angles of the hip and knee joints, respectively, at the i point in the movement cycle. The CRP patterns were further summarized using mean absolute relative phase (MARP) (Kurz et al., 2005) and deviation phase (DP) (Hamill et al., 1999). Lower MARP values indicate coordination closer to in-phase, whereas higher MARP values indicate coordination closer to anti-phase. DP represents the magnitude of coordination variability; higher DP indicates greater variability but does not, by itself, imply better or poorer motor control.

### Statistical analysis

All statistical analyses were performed using custom MATLAB scripts, with participant-level values used for between-group comparisons. Normality and homogeneity of variance were assessed using the Shapiro–Wilk and Levene’s tests, respectively. Depending on data distribution and variance homogeneity, independent-samples t-tests, Welch’s t-tests, or Mann–Whitney U tests were used. CoA was summarized using circular mean ± circular SD and compared using exact two-sided circular permutation tests. MARP and DP were compared between groups within each movement phase. Pointwise CRP comparisons across the 500 normalized time points were exploratory and were not corrected for multiple testing. Appropriate effect sizes were reported for between-group comparisons. Statistical significance was set at p < 0.05, and reported p values were unadjusted. Benjamini–Hochberg FDR correction was additionally performed as a sensitivity analysis for the 11 comparisons in **Table 1**.

**Table 1:** Temporal characteristics of muscle synergies and co-contraction indices of the kicking and supporting limbs during the Tai Chi heel-kick movement in the Master-level and first-class groups.

Variable	Module / limb region	Master-level group mean ± SD	First-class group mean ± SD	Effect estimate	*p*
Muscle synergy number					
Participant-level mean *N_syn_*	—	3.73 ± 0.37	3.63 ± 0.33	*r*_rb_ = 0.203	0.499^a^
Module-specific temporal parameters					
CoA, % cycle	M1	29.51 ± 10.57	56.97 ± 11.89	Δ_circ_ = -27.46%	0.004^b^
FWHM, % cycle	M1	2.11 ± 4.59	0.19 ± 0.42	*r*_rb_ = 0.400	0.274^a^
CoA, % cycle	M2	97.69 ± 10.17	2.34 ± 12.95	Δ_circ_ = -4.65%	0.556^b^
FWHM, % cycle	M2	1.70 ± 2.22	1.08 ± 0.85	*r*_rb_ = 0.028	1.000^a^
CoA, % cycle	M3	54.72 ± 3.88	53.24 ± 6.04	Δ_circ_ = 1.48%	0.645^b^
FWHM, % cycle	M3	4.98 ± 11.49	0.08 ± 0.12	*r*_rb_ = 0.111	0.775^a^
Co-contraction characteristics					
Co-contraction index	Supporting-limb thigh	23.19 ± 5.18	22.04 ± 10.65	Hedges’ *g* = 0.127	0.817^c^
Co-contraction index	Supporting-limb shank	8.25 ± 2.67	10.35 ± 8.01	*r*_rb_ =0.056	0.936^a^
Co-contraction index	Kicking-limb thigh	34.44 ± 13.95	23.57 ± 12.07	Hedges’ *g* = 0.769	0.180^c^
Co-contraction index	Kicking-limb shank	36.66 ± 11.60	30.45 ± 4.43	Hedges’ *g* = 0.653	0.249^c^

^a^Mann–Whitney U test; ^b^exact two-sided circular permutation test; ^c^independent-samples t-test.

## Results

### Estimated spinal segmental motor output characteristics

As shown in **Figure 2**, the Master-level and first-class groups exhibited different spatiotemporal patterns of lumbosacral motor output during the Tai Chi heel-kick movement cycle. **Figure 2A** shows that the most apparent group differences occurred in the S3 and S1 segments. In the first-class group, S3 output increased rapidly during the early portion of the movement cycle and reached its peak earlier, whereas in the Master-level group, S3 output increased more gradually and remained relatively high during the middle and later portions of the cycle. S1 showed a different temporal pattern: the Master-level group reached its peak earlier, whereas the peak occurred later in the first-class group. In comparison, the mean temporal profiles of S2, L4, and L5 were generally similar between the two groups.

**Figure 2:**
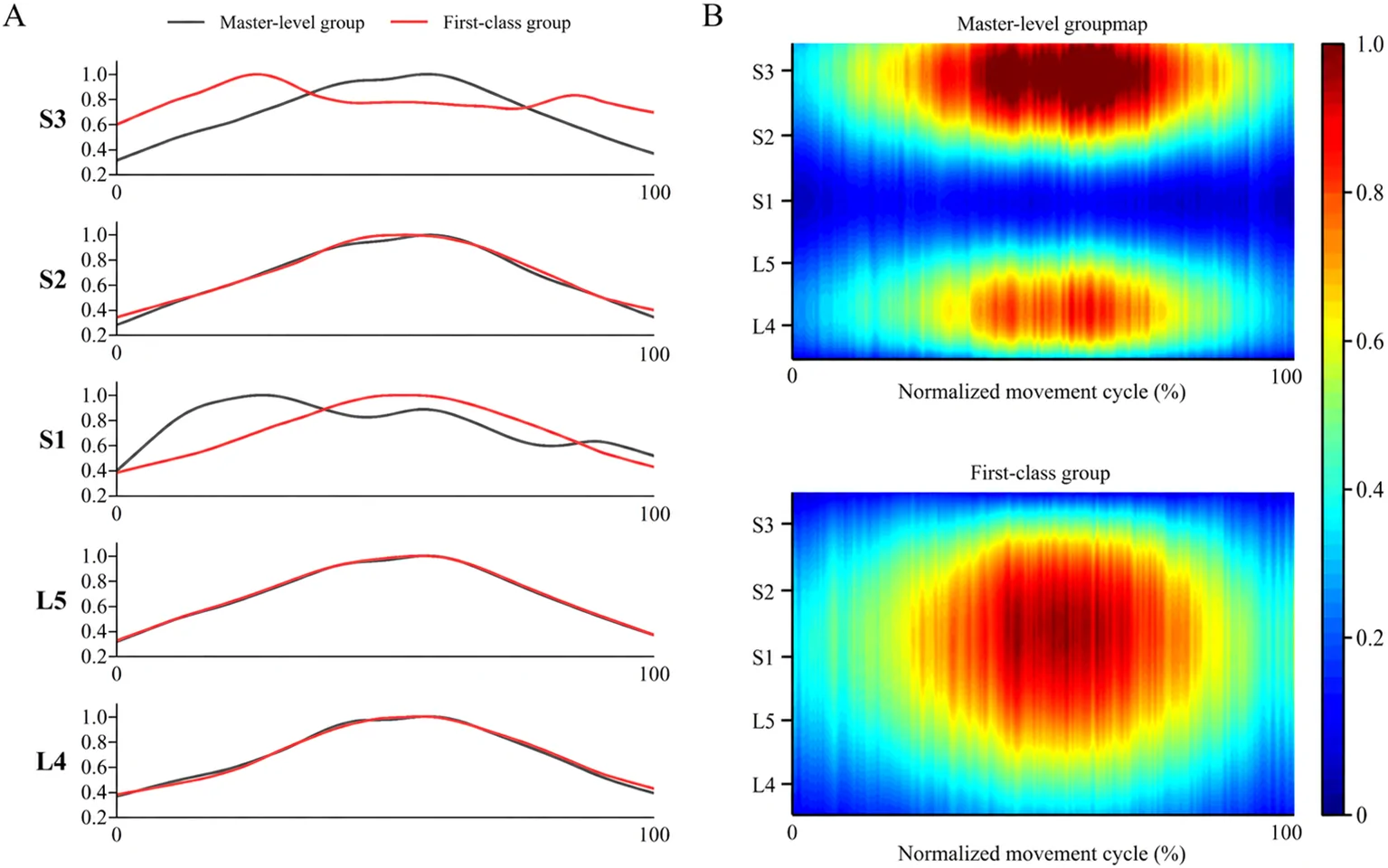
Descriptive group-average patterns of estimated spinal segmental motor output during the Tai Chi heel-kick movement. **(A)** Group-average temporal profiles of estimated motoneuron-pool output across the L4–S3 spinal segments in the Master-level and first-class groups. **(B)** Group-average rostrocaudal output maps for the Master-level group (upper) and first-class group (lower). The horizontal axis represents the normalized movement cycle from 0% to 100%. Values in both contour maps were normalized to the range 0–1, and the same color scale was used for both groups to facilitate direct visual comparison.

The spatiotemporal heatmaps in **Figure 2B** further showed that the higher output in the Master-level group was mainly concentrated in the S3 and L4 regions during the middle-to-late portions of the movement cycle, forming two relatively separate regions of high output. In contrast, the higher output in the first-class group was mainly distributed around S1–S2 and formed a broader, continuous region of elevated output during the middle portion of the movement cycle.

### Muscle synergy number and group-level representative modules

The number of muscle synergies extracted from each analyzed trial for each participant is presented in **Supplementary Table 1**. Trial-level values were first averaged within each participant before the between-group comparison. The participant-level mean was 3.73 ± 0.37 in the Master-level group and 3.63 ± 0.33 in the first-class group. No statistically significant difference was observed, indicating that the dimensionality required to reconstruct the heel-kick EMG patterns was broadly comparable between groups.

Within-group clustering of the muscle-weighting vectors extracted from all trials identified three representative group-level synergy modules in each group. Because cluster labels do not have an inherent correspondence across independently clustered groups, the group-average muscle-weighting vectors were matched one-to-one using cosine similarity. Master-level original M1 was matched with first-class original M3, with the highest cosine similarity of 0.991. Master-level original M2 was matched with first-class original M2, with a cosine similarity of 0.962, and Master-level original M3 was matched with first-class original M1, with a cosine similarity of 0.889. Based on these matching results, the first-class modules in **Figure 3** were reordered, and the matched modules were consistently labelled M1, M2, and M3.

**Figure 3:**
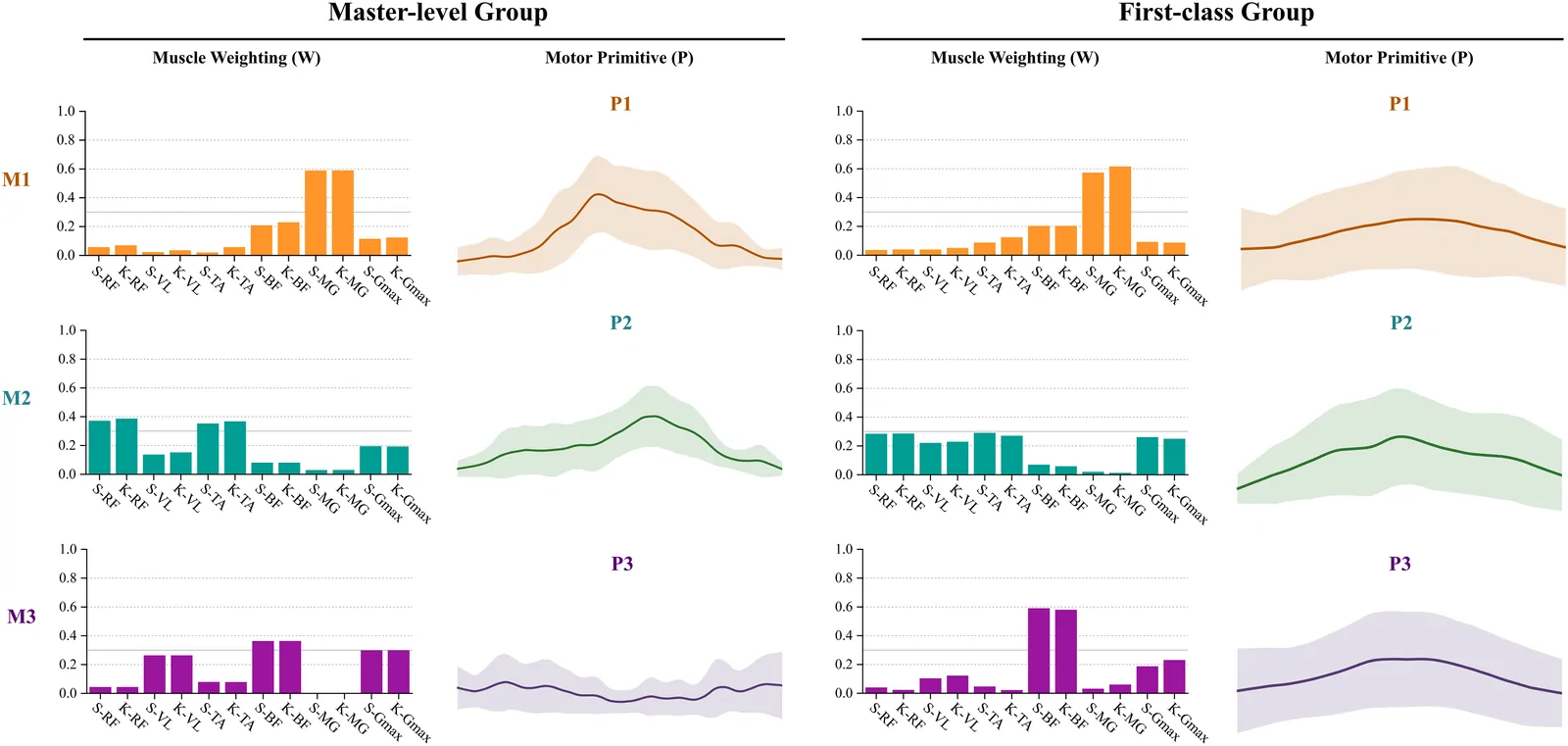
Matched muscle synergy characteristics during the Tai Chi heel-kick movement. M1–M3 represent the spatial structures of the three matched group-level representative synergy modules, expressed as muscle-weighting vectors (W). P1–P3 represent the corresponding normalized temporal activation patterns. Lines represent group-average activation profiles and shaded regions represent between-participant variability. The right limb was defined as the kicking limb and the left limb as the supporting limb.

As shown in **Figure 3**, matched M1 was characterized in both groups by high bilateral medial gastrocnemius weightings, accompanied by secondary contributions from the bilateral biceps femoris, whereas the gluteus maximus and the remaining muscles showed relatively lower weightings. The M1 muscle-weighting distributions were generally similar between groups, consistent with the high similarity between the matched cluster centers. The corresponding motor primitive, P1, exhibited a unimodal activation pattern that gradually increased during the middle portion of the movement cycle and subsequently declined. However, the mean activation profile was broader in the first-class group, with greater inter-individual variability.

Matched M2 involved the combined contribution of the rectus femoris, tibialis anterior, and gluteus maximus in both groups. In the Master-level group, M2 was mainly characterized by bilateral rectus femoris and tibialis anterior weightings, with secondary contributions from the gluteus maximus. In the first-class group, the muscle contributions were more broadly distributed, with noticeable involvement of the bilateral vastus lateralis and gluteus maximus in addition to the rectus femoris and tibialis anterior. The corresponding P2 profiles gradually increased from the early portion of the movement cycle, reached higher activation during the middle-to-late portion, and subsequently declined in both groups, indicating a generally similar overall temporal activation pattern.

Matched M3 in the Master-level group was mainly composed of the bilateral biceps femoris, vastus lateralis, and gluteus maximus. In the first-class group, M3 was predominantly characterized by the bilateral biceps femoris, accompanied by contributions from the gluteus maximus and smaller contributions from the vastus lateralis and tibialis anterior. Compared with M1 and M2, M3 showed a lower between-group cosine similarity, indicating a more apparent difference in the muscle-weighting composition of this module. The mean P3 profiles also showed different descriptive patterns: the Master-level group exhibited a lower-amplitude and more fluctuating activation profile, whereas the first-class group showed a broader increase in activation during the middle portion of the movement cycle. These differences in the motor primitives are reported descriptively, while the module-specific statistical comparisons of CoA and FWHM are presented in the following subsection.

### Module-specific CoA and FWHM

A significant difference between-groups was observed in the center of activity of M1. The circular mean CoA of M1 was 29.51 ± 10.57% of the movement cycle in the Master-level group and 56.97 ± 11.89% in the first-class group. The absolute circular mean difference was 27.46% of the movement cycle (exact circular permutation test, unadjusted p=0.004). This result indicates that the primary activation center of M1 occurred earlier in the Master-level group than in the first-class group. After a Benjamini–Hochberg sensitivity correction for the 11 comparisons included in **Table 1**, this result remained below the FDR threshold ( q=0.048). One participant in the first-class group had no synergy assigned to this matched module; therefore, the M1 analysis included six participants in the Master-level group and five participants in the first-class group.

M1 FWHM did not differ significantly between the Master-level and first-class groups, with values of 2.11 ± 4.59% and 0.19 ± 0.42% of the movement cycle, respectively ( p=0.274). No significant difference between groups was observed in M2 CoA. Although the circular mean values were 97.69 ± 10.17% in the Master-level group and 2.34 ± 12.95% in the first-class group, both values were located close to the boundary between the end and the beginning of the normalized movement cycle. The actual circular mean difference was therefore only 4.65% of the movement cycle ( p=0.556) and should not be interpreted using ordinary linear subtraction. M2 FWHM also did not differ significantly between groups (1.70 ± 2.22% vs. 1.08 ± 0.85%; p=1.000).

The circular mean CoA of M3 was 54.72 ± 3.88% of the movement cycle in the Master-level group and 53.24 ± 6.04% in the first-class group, with no significant between-group difference (circular mean difference = 1.48% of the movement cycle, p=0.645). No significant group difference was observed in M3 FWHM either (4.98 ± 11.49% vs. 0.08 ± 0.12%; p=0.775). Therefore, the between-group difference in temporal characteristics was mainly restricted to the activation center of M1 and did not represent a general change in the activation duration of all modules.

### Joint coordination characteristics

As shown in **Figure 4**, exploratory uncorrected pointwise comparisons of the continuous relative phase curves suggested potential between-group separation in selected portions of the movement cycle. Specifically, the supporting-limb hip–knee CRP (**Figure 4A**) and kicking-limb hip–knee CRP (**Figure 4C**) showed group differences during distinct portions of the movement cycle (19–51% in **Figure 4A** and 98–100% in **Figure 4C**). In contrast, no significant group differences were observed in the supporting-limb knee–ankle CRP (**Figure 4B**) or kicking-limb knee–ankle CRP (**Figure 4D**). These findings indicate that the observed group differences were concentrated in hip–knee coordination rather than knee–ankle coordination.

**Figure 4:**
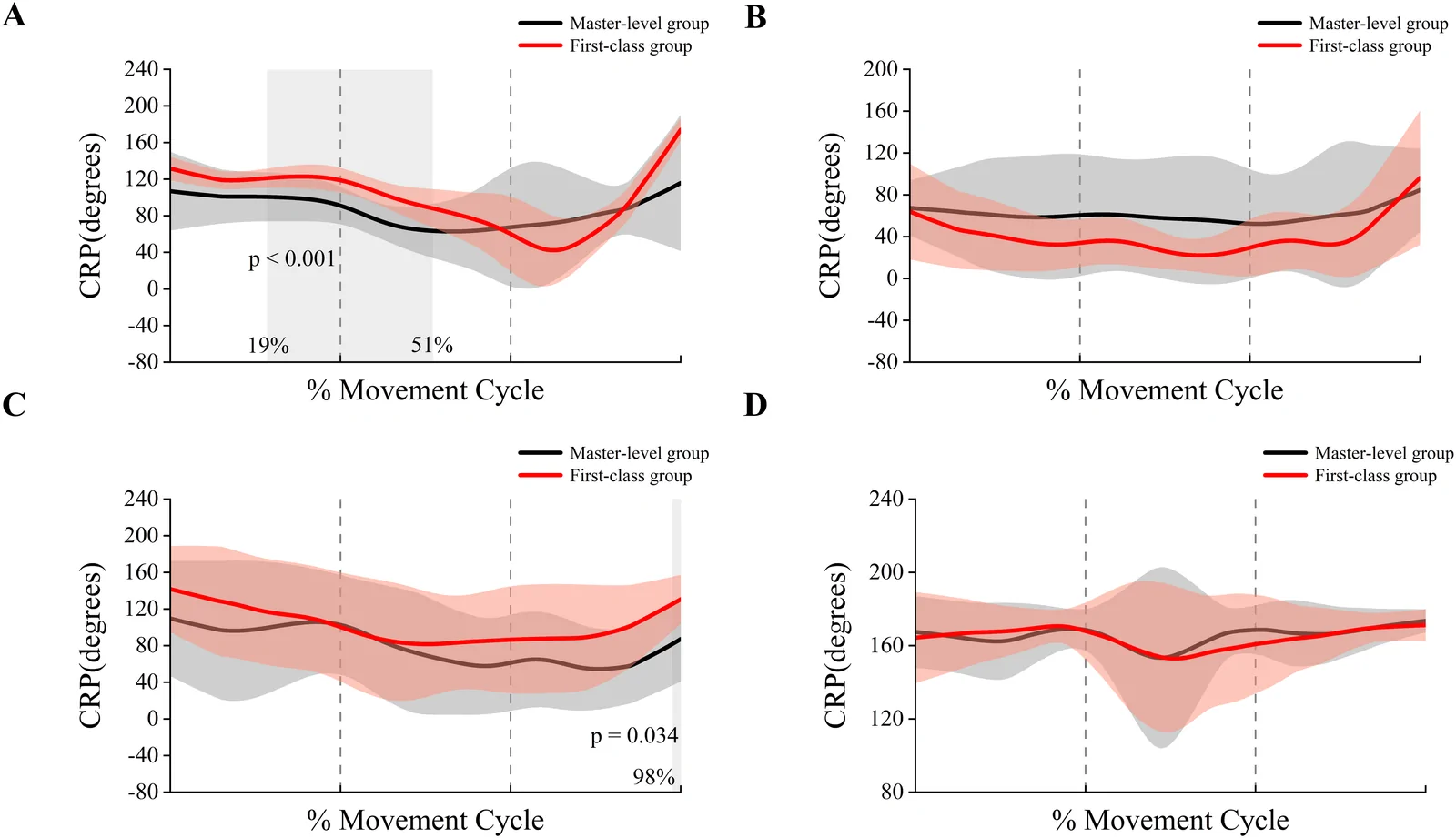
Continuous relative phase (CRP) during the Tai Chi heel-kick movement. **(A)** Supporting-limb hip–knee CRP. **(B)** Supporting-limb knee–ankle CRP. **(C)** Kicking-limb hip–knee CRP. **(D)** Kicking-limb knee–ankle CRP. The right limb was standardized as the kicking limb and the left limb as the supporting limb. The shaded intervals represent exploratory uncorrected pointwise comparisons and should not be interpreted as confirmatory significance intervals.

The CRP-derived MARP and DP results are presented in **Table 2**. Lower MARP values indicate a more in-phase coordination pattern between the two joints, whereas higher MARP values indicate a coordination pattern closer to anti-phase. Lower DP values indicate lower coordination variability, whereas higher DP values indicate greater coordination variability.

**Table 2:** Statistical analysis results of MARP and DP.

Period	Joint pair	MARP				DP			
		MLG	FCG	d	p	MLG	FCG	d	p
Knee-lift phase	Supporting-limb hip–knee	120.35 ± 82.54	169.80 ± 8.93	−0.842^###^	0.111	3.21 ± 1.57	4.71 ± 2.86	−0.650^##^	0.187
	Supporting-limb knee–ankle	60.90 ± 67.53	38.26 ± 27.21	0.440^#^	0.372	10.50 ± 5.87	14.27 ± 8.40	−0.521^##^	0.286
	Kicking-limb hip–knee	118.42 ± 67.38	126.20 ± 54.06	−0.127	0.790	12.32 ± 13.78	9.60 ± 4.03	0.268^#^	0.583
	Kicking-limb knee–ankle	157.35 ± 25.55	175.42 ± 2.68	−0.995^###^	0.067	13.67 ± 16.59	2.99 ± 1.90	0.905^###^	0.090
Push-off phase	Supporting-limb hip–knee	75.76 ± 51.73	151.88 ± 1.70	−2.080^###^	0.002**	17.92 ± 8.63	10.87 ± 1.60	1.136^###^	0.041*
	Supporting-limb knee–ankle	61.05 ± 59.76	21.66 ± 17.00	0.897^###^	0.089	10.37 ± 4.39	10.07 ± 4.93	0.064	0.894
	Kicking-limb hip–knee	90.20 ± 43.43	81.32 ± 20.34	0.262^#^	0.589	30.86 ± 13.81	28.87 ± 8.54	0.173	0.719
	Kicking-limb knee–ankle	159.90 ± 10.15	155.67 ± 20.00	0.266^#^	0.580	18.45 ± 14.17	17.89 ± 15.70	0.037	0.938
Recovery phase	Supporting-limb hip–knee	95.65 ± 16.06	110.54 ± 18.57	−0.858^###^	0.088	22.35 ± 1.98	20.86 ± 1.43	0.863^###^	0.086
	Supporting-limb knee–ankle	62.70 ± 62.04	20.38 ± 10.12	0.952^###^	0.076	17.21 ± 5.19	13.12 ± 7.22	0.651^##^	0.186
	Kicking-limb hip–knee	22.45 ± 15.49	107.64 ± 47.72	−2.402^###^	<0.001***	11.50 ± 7.50	26.34 ± 15.24	−1.236^###^	0.018*
	Kicking-limb knee–ankle	170.49 ± 5.16	173.60 ± 3.94	−0.677^##^	0.170	5.05 ± 3.08	4.31 ± 4.70	0.187	0.697

Values are presented as mean ± standard deviation. MLG, master -level group; FCG, first-class group; MARP, mean absolute relative phase; DP, deviation phase. Effect sizes are reported as Cohen’s d. “#” denotes a small effect size (0.2 ≤ |d| < 0.5), “##” denotes a medium effect size (0.5 ≤ |d| < 0.8), and “###” denotes a large effect size (|d| ≥ 0.8). “*” indicates p < 0.05, “**” indicates p < 0.01, and “***” indicates p < 0.001.

During the knee-lift phase, no significant between-group differences were observed in MARP or DP for any joint pair. Nevertheless, large effect sizes were observed for supporting-limb hip–knee MARP, kicking-limb knee–ankle MARP, and kicking-limb knee–ankle DP. The MLG tended to show lower MARP in the supporting-limb hip–knee and kicking-limb knee–ankle joint pairs, suggesting a possible tendency toward a more in-phase coordination pattern. However, these differences did not reach statistical significance and should therefore be interpreted cautiously.

During the push-off phase, significant between-group differences were found in the supporting-limb hip–knee joint. The MLG showed a significantly lower MARP than the FCG (75.76 ± 51.73° vs. 151.88 ± 1.70°, d = −2.080, p = 0.002), indicating a more in-phase supporting-limb hip–knee coordination pattern during this force-generation phase. However, the MLG also showed a significantly higher DP than the FCG (17.92 ± 8.63° vs. 10.87 ± 1.60°, d = 1.136, p = 0.041), indicating greater coordination variability. Therefore, during the push-off phase, high-level practitioners exhibited a more in-phase but more variable supporting-limb hip–knee coordination pattern. No significant group differences were observed in the remaining joint pairs during this phase.

During the recovery phase, significant between-group differences were observed in the kicking-limb hip–knee joint. The MLG showed significantly lower MARP than the FCG (22.45 ± 15.49° vs. 107.64 ± 47.72°, d = −2.402, p < 0.001), indicating a more in-phase kicking-limb hip–knee coordination pattern. The MLG also showed significantly lower DP than the FCG (11.50 ± 7.50° vs. 26.34 ± 15.24°, d = −1.236, p = 0.018), indicating lower coordination variability during recovery. The supporting-limb hip–knee joint showed large effect sizes for both MARP and DP, but the differences were not statistically significant. No significant between-group differences were observed in knee–ankle coordination during the recovery phase.

## Discussion

The present study examined neuromuscular control during the Tai Chi heel-kick movement in practitioners with different levels of motor experience by integrating estimated spinal segmental motor output, muscle synergy analysis, lower-limb antagonist co-contraction, and joint coordination. The principal findings were as follows. First, the Master-level and first-class groups showed visually distinct temporal and rostrocaudal patterns of estimated lumbosacral motor output, although these patterns were descriptive rather than inferentially confirmed. Second, the participant-level number of extracted synergies was comparable between groups, while the matched group-level modules differed in their muscle-weighting composition and temporal organization. In particular, the activation center of the medial-gastrocnemius-dominant M1 occurred earlier in the Master-level group, whereas no general between-group difference was observed in module activation duration. Third, none of the four co-contraction indices differed significantly between groups. Finally, the most robust coordination differences were phase- and joint-specific: the Master-level group showed a more in-phase but more variable supporting-limb hip-knee pattern during push-off and a more in-phase and less variable kicking-limb hip-knee pattern during recovery.

The estimated spinal-output profiles provide a preliminary view of how the recorded lower-limb muscle activity was distributed across lumbosacral segments over the normalized movement cycle. At S3, the first-class group showed a more rapid early increase and an earlier peak, whereas the Master-level group exhibited a more gradual rise and maintained relatively high output during the middle and later portions of the cycle. At S1, the Master-level group peaked earlier, while the first-class group showed a later peak. The heatmaps further suggested that the Master-level group had relatively high output in the S3 and L4 regions during the middle-to-late portion of the movement cycle, whereas the first-class group showed a broader region of elevated output centered around S1-S2. These observations may reflect differences in the relative timing of the muscles contributing to the segmental reconstruction. Under the Kendall-based mapping used in this study, the estimated sacral output was influenced mainly by the recorded muscles assigned to S1-S3, particularly the medial gastrocnemius, whereas L4-L5 predominantly reflected contributions from the quadriceps muscles and tibialis anterior. Previous studies have shown that muscle synergies and the temporal and rostrocaudal organization of spinal motor output can vary across motor tasks, developmental stages, pathological conditions, and training-related contexts (Cheung et al., 2005, 2009; Dominici et al., 2011; Grasso et al., 2004; Ivanenko et al., 2008, 2013; Monaco et al., 2010; Taube et al., 2008). However, the present profiles were reconstructed indirectly by projecting surface EMG activity onto anatomical distributions of spinal motoneuron pools (Yakovenko et al., 2002), and no participant-level inferential tests were applied to these curves. They should therefore be interpreted as descriptive muscle-based estimates rather than direct evidence of experience-related spinal reorganization.

The synergy-number results indicate that the two groups used a broadly comparable dimensionality of muscle activation to perform the heel-kick movement. The absence of a significant difference in participant-level synergy number suggests that greater motor experience was not expressed through a simple reduction or increase in the number of modules required to reconstruct the EMG signals. Instead, the more informative differences were found after matching the independently clustered group-level modules. M1 and M2 showed high cross-group cosine similarity, whereas M3 showed a lower, although still substantial, similarity. This pattern suggests that the basic modular structure of the movement was shared across groups, while motor experience may have been associated with the reweighting of particular muscles within specific modules. Such an interpretation is consistent with evidence that skill learning can be expressed through the selection and tuning of muscle synergies (Kargo and Nitz, 2003) and with the broader use of synergy analysis as a low-dimensional description of motor function (Ting and McKay, 2007). Nevertheless, NMF-derived synergies remain analytical decompositions of correlated EMG activity and should not be interpreted as direct neural modules (Tresch and Jarc, 2009). Accordingly, terms such as greater economy or neural efficiency cannot be inferred from the present weighting patterns alone.

The module-specific temporal analysis further refines this interpretation. Matched M1 was characterized mainly by high bilateral medial gastrocnemius weightings, with secondary contributions from the bilateral biceps femoris. Its circular mean CoA occurred substantially earlier in the Master-level group than in the first-class group, and this difference remained below the Benjamini-Hochberg FDR threshold in the sensitivity analysis. This finding suggests an experience-related temporal advance in the principal activation center of a posterior lower-limb module involving plantarflexor and knee-flexor contributions. Such an earlier activation center may facilitate the timely organization of support, force transfer, or transition between movement phases, although the present data do not identify the specific biomechanical function responsible for the shift. In contrast, the CoA of M2 and M3 did not differ significantly between groups, and no module showed a significant between-group difference in FWHM. Thus, the temporal effect was module-specific rather than a generalized shift in activation timing or duration. Because CoA is a circular descriptor, values near 0% and 100% represent adjacent points in the movement cycle rather than opposite temporal positions (Berens, 2009). The M2 values illustrate this issue and support the use of circular, rather than ordinary linear, interpretation.

The spatial structure of the remaining modules also supports a selective rather than global experience-related difference. M2 involved contributions from the rectus femoris, tibialis anterior, and gluteus maximus in both groups, with a broader distribution of weightings in the first-class group. M3 included substantial biceps femoris contributions in both groups, but the Master-level pattern also showed greater involvement of the vastus lateralis and gluteus maximus. The lower cross-group similarity of M3 indicates that this module displayed the largest descriptive difference in muscle composition. However, the corresponding CoA and FWHM results were not significant, and the visual differences in the motor primitives were not subjected to point-by-point inferential testing. These findings therefore support experience-related differences in the organization of muscle contributions within selected modules, but they do not support a general conclusion that Master-level practitioners used uniformly sharper, more concentrated, or more economical activation patterns.

The co-contraction results provide an additional constraint on the interpretation of skill-related muscle organization. None of the supporting- or kicking-limb thigh and shank co-contraction indices differed significantly between groups. Therefore, the present data do not show that greater experience was accompanied by a generalized reduction or increase in antagonist co-contraction. The kicking-limb thigh and shank indices were numerically higher in the Master-level group and were associated with moderate-to-large effect estimates, but the corresponding comparisons were not statistically significant. These numerical differences may warrant further investigation in a larger sample, particularly because co-contraction can contribute both to joint stiffness and to movement control under single-leg support. At present, however, they should be treated as exploratory rather than as evidence of a distinct experience-related co-contraction strategy.

The joint coordination findings showed a clearer phase-specific pattern. During push-off, the Master-level group demonstrated a lower supporting-limb hip-knee MARP, indicating a more in-phase coordination pattern, together with a higher DP, indicating greater coordination variability. Greater variability in this context should not automatically be interpreted as poorer control. It may reflect a broader range of joint solutions used to regulate force generation and balance during the mechanically demanding push-off phase. During recovery, the Master-level group showed both lower kicking-limb hip-knee MARP and lower DP, indicating a more in-phase and less variable coordination pattern as the kicking limb was withdrawn and postural control was re-established. These findings show that expertise was not associated with a single global coordination strategy; instead, coordination organization differed according to the functional demands of each phase. This interpretation is consistent with the view that skilled movement depends on organizing multiple degrees of freedom into task-specific patterns (Turvey, 1990). with relative-phase measures providing information about both coordination mode and variability (Miller et al., 2010) (Wilson et al., 2008). The pointwise CRP intervals should be interpreted cautiously because they were derived from exploratory comparisons across 500 normalized time points without correction for multiple testing; the phase-level MARP and DP analyses therefore provide the primary basis for inferential interpretation.

The concentration of statistically robust differences at the hip-knee joints is also consistent with findings from kicking-based martial arts. Kim et al. emphasized the contribution of hip-knee inter-joint coordination to kicking velocity in taekwondo techniques (Kim et al., 2011). Falco et al. showed that expert and novice taekwondo athletes differed in execution time and impact force under different distance conditions (Falco et al., 2009), while Gavagan and Sayers reported discipline-specific kinematic organization among skilled Muay Thai, karate, and taekwondo practitioners (Gavagan and Sayers, 2017). The present results extend this general expertise-related framework to the Tai Chi heel-kick, but the task constraints are different from those of high-speed combat kicks. The Tai Chi movement requires controlled single-leg support, gradual force production, maintenance of trunk orientation, and stable recovery. The more in-phase supporting-limb hip-knee pattern during push-off and the more consistent kicking-limb hip-knee pattern during recovery may therefore reflect adaptations for combining controlled limb extension with dynamic balance rather than for maximizing impact velocity.

The distal knee-ankle results should be interpreted more conservatively. No statistically significant knee-ankle CRP differences were observed, and the phase-level MARP and DP comparisons were also non-significant. Several variables nevertheless showed moderate-to-large effect sizes, particularly during the knee-lift phase. It would therefore be inappropriate to conclude that distal coordination was identical between groups. A more balanced interpretation is that the current sample provided stronger evidence for proximal hip-knee differences, whereas possible knee-ankle differences remain uncertain. This view is compatible with dynamic-systems approaches, in which variability and effect magnitude can reveal potentially meaningful changes in movement organization even when conventional significance thresholds are not reached (Stergiou and Decker, 2011). CRP-based analyses have similarly been used to distinguish coordination patterns in clinical and biomechanical settings, although their interpretation depends strongly on task constraints, analysis choices, and sample characteristics (Lamb and Stöckl, 2014).

Taken together, the findings support a multilevel but non-hierarchical interpretation of heel-kick expertise. The descriptive segmental-output patterns, the matched synergy structures, the earlier CoA of M1, and the phase-specific hip-knee coordination differences all point to experience-related changes in when and how selected lower-limb muscles and joints are organized. However, the present study does not demonstrate a causal sequence from spinal output to muscle synergy and then to joint coordination. Long-term training has been associated with changes in the modular organization of balance control (Sawers et al., 2015), and experimental evidence suggests that muscle synergies may have central representations while remaining strongly shaped by biomechanical and task constraints (Overduin et al., 2015). Within this framework, skilled Tai Chi heel-kick performance may depend on the coordinated integration of selective muscle recruitment, phase-appropriate proximal joint organization, and postural regulation rather than on a simple reduction in synergy number or co-contraction.

These results have several practical implications. Training should emphasize the temporal organization of the entire movement rather than the final kicking posture alone. During knee lift and transition into push-off, practitioners may benefit from drills that develop timely recruitment of the posterior lower-limb musculature while maintaining stable single-leg support. During push-off, coaches should focus on coordinated supporting-limb hip-knee motion while allowing sufficient flexibility in joint coordination to accommodate balance and force-generation demands. During recovery, practice should emphasize synchronized withdrawal of the kicking limb and consistent re-control of the center of mass. Slow segmented practice, progressive linking of movement phases, and video feedback may help practitioners identify discontinuities in these transitions. Surface EMG feedback, where available, may be useful for individualized assessment of recruitment timing or excessive co-contraction, but the present findings do not support prescribing a uniform reduction in co-contraction for all practitioners.

Several limitations should be considered. The sample was small and included only male practitioners, limiting statistical power and generalizability. The segmental motor-output profiles were indirect estimates derived from a restricted set of surface EMG recordings and anatomical innervation maps, and they were not evaluated with participant-level inferential statistics. The NMF results and cross-group module matching depended on analytical choices, including normalization, rank selection, clustering, and cosine-similarity matching. Although the M1 CoA result remained below the FDR threshold in a sensitivity analysis, multiple variables and movement phases were examined, and the remaining unadjusted comparisons should be interpreted cautiously. The cross-sectional design also prevents causal inference regarding training effects. Future studies should include larger and more diverse samples, longitudinal training designs, a broader set of lower-limb muscles, and direct statistical evaluation of the segmental and time-varying synergy profiles.

## Conclusion

This study examined neuromuscular control during the Tai Chi heel-kick movement in practitioners with different levels of motor experience. The two groups showed descriptive differences in the temporal and rostrocaudal distribution of estimated lumbosacral motor output, while the participant-level number of muscle synergies was comparable. Experience-related differences were more evident in the composition and timing of selected synergy modules, particularly the earlier activation center of matched M1 in the Master-level group, rather than in a generalized change in synergy number, activation duration, or antagonist co-contraction. Joint coordination differences were also phase- and joint-specific, with distinct supporting-limb and kicking-limb hip–knee coordination patterns during push-off and recovery. These findings suggest that skilled heel-kick performance is associated with selective, task-dependent organization of lower-limb muscle recruitment and proximal joint coordination.

## Data Availability

The raw data supporting the conclusions of this article will be made available by the authors, without undue reservation.
