# Supplementary material for: Neuromuscular control strategies during the Tai Chi heel-kick movement across different levels of motor experience
Source: Front Physiol. 2026 Sep 17;17:1873507. doi: 10.3389/fphys.2026.1873507 (PMC13626890; doi:10.3389/fphys.2026.1873507)
Supplement: Supplementary file 1 [file Table1.docx]

# Supplementary Table S1: Number of muscle synergies extracted from each analyzed trial for each participant in both groups

| Group | Participant ID | Trial 1 | Trial 2 | Trial 3 | Trial 4 | Trial 5 | Mean ± SD | Range |
| --- | --- | --- | --- | --- | --- | --- | --- | --- |
| Master-level | 1TH | 4 | 4 | 3 | 4 | 3 | 3.60 **±** 0.55 | 3–4 |
|  | 2TH | 4 | 3 | 4 | 3 | 3 | 3.40 **±** 0.55 | 3–4 |
|  | 3TH | 4 | 3 | 5 | 4 | 2 | 3.60 **±** 1.14 | 2–5 |
|  | 4TH | 4 | 3 | 4 | 4 | 3 | 3.60 **±** 0.55 | 3–4 |
|  | 5TH | 4 | 5 | 3 | 2 | 4 | 3.60 **±** 1.14 | 2–5 |
|  | 6TH | 4 | 4 | 2 | 4 | 4 | 3.60 **±** 0.89 | 2–4 |
|  | 7TH | 4 | 3 | 3 | 5 | 4 | 3.80 **±** 0.84 | 3–5 |
|  | 8TH | 4 | 6 | 6 | 4 | 3 | 4.60 **±** 1.34 | 3–6 |
| First-class | 9TH | 4 | 3 | 5 | 3 | 6 | 4.20 **±** 1.30 | 3–6 |
|  | 10TH | 4 | 3 | 3 | 4 | 2 | 3.20 **±** 0.84 | 2–4 |
|  | 11TH | 4 | 3 | 5 | 4 | 4 | 4.00 **±** 0.71 | 3–5 |
|  | 12TH | 5 | 3 | 4 | 2 | 3 | 3.40 **±** 1.14 | 2–5 |
|  | 13TH | 4 | 2 | 5 | 3 | 4 | 3.60 **±** 1.14 | 2–5 |
|  | 14TH | 3 | 3 | 4 | 4 | 4 | 3.60 **±** 0.55 | 3–4 |
|  | 15TH | 3 | 4 | 3 | 3 | 4 | 3.40 **±** 0.55 | 3–4 |
|  | 16TH | 4 | 2 | 5 | 4 | 3 | 3.60 **±** 1.14 | 2–5 |

**Note:** denotes the number of muscle synergies selected at the individual-trial level. Participant-level means and standard deviations were calculated across the five analyzed trials. For the between-group comparison, trial-level values were first averaged within each participant to avoid treating repeated trials as independent observations. The participant-level mean was 3.73 ± 0.37 in the Master-level group and 3.63 ± 0.33 in the first-class group. No significant between-group difference was observed (Mann–Whitney U=38.5, unadjusted p=0.499, rank-biserial r=0.203).
